# Physical activity and quality of life in statin users

**DOI:** 10.1093/fampra/cmaf080

**Published:** 2025-10-21

**Authors:** Mert Er, Ogulcan Come, Gizem Limnili, Nilgün Özçakar

**Affiliations:** Department of Family Medicine, Dokuz Eylül University Faculty of Medicine, Izmir 35330, Turkey; Department of Family Medicine, Dokuz Eylül University Faculty of Medicine, Izmir 35330, Turkey; Department of Family Medicine, Dokuz Eylül University Faculty of Medicine, Izmir 35330, Turkey; Department of Family Medicine, Dokuz Eylül University Faculty of Medicine, Izmir 35330, Turkey

**Keywords:** statins, physical activity, quality of life, dyslipidemia, muscle symptoms, primary care

## Abstract

**Background:**

Dyslipidemia is a major risk factor for cardiovascular disease, with statins widely prescribed to manage lipid levels. However, statin-associated muscle symptoms and the interplay with physical activity (PA) may impact patients' quality of life (QoL). This study aimed to explore the relationship between PA levels and QoL in statin users.

**Methods:**

A cross-sectional study was conducted among 384 adult statin users attending Dokuz Eylül University Family Health Centers. PA was assessed using the International Physical Activity Questionnaire, and QoL was evaluated with the Short Form-36 Health Survey. Due to the non-normal data distribution, nonparametric statistical tests were applied.

**Results:**

Of the participants, 18.2% were inactive, 65.1% minimally active, and 16.7% very active. Higher PA levels were associated with significantly better scores in physical functioning, role limitations due to physical health, energy/fatigue, social functioning, pain, and general health (*P* < 0.001). Sitting duration was negatively correlated with most Short Form-36 domains. No significant association was found between PA and role limitations due to emotional problems.

**Conclusion:**

Higher physical activity levels are positively associated with better quality of life among statin users, despite potential concerns regarding muscle symptoms. Regular, moderate physical activity may enhance this population's physical and mental health outcomes.

Key messages

- Physical activity is positively associated with quality of life in statin users.
- Higher activity levels improve physical, social, and general health domains.
- Sitting time shows negative correlations with most quality of life measures.
- Quality of life benefits were observed across multiple activity levels.
- Findings support encouraging regular activity despite statin-related concerns.

## Introduction

Dyslipidemia, defined by abnormal levels of lipids in the bloodstream, is a well-established modifiable risk factor for atherosclerotic cardiovascular diseases (ASCVD). Its prevalence continues to rise globally, influenced by genetic predispositions and environmental factors [1]. The increasing adoption of unhealthy lifestyle habits has significantly contributed to this growing burden. In 2019, 3.78 million deaths worldwide due to ischemic heart disease were attributed to high low-density lipoprotein (LDL) cholesterol levels (LDL-C) levels, accounting for 44.3% of these deaths. Addressing cardiovascular risk factors, including those related to dyslipidemia through dietary modifications and pharmacological treatment, as well as broader lifestyle measures such as regular exercise and smoking cessation, remains crucial for the primary and secondary prevention of cardiovascular events [2, 3].

There are different treatments for existing dyslipidemia, including statins (HMG-CoA reductase inhibitors), which have revolutionized the management of lipid disorders. By competitively inhibiting the rate-limiting step in cholesterol biosynthesis, statins effectively reduce LDL-C levels and, as a result, have become a cornerstone therapy in primary and secondary prevention of coronary atherosclerosis [4]. Although generally well-tolerated, statins do not come without adverse effects. One of the most reported and clinically significant side effects is statin-associated muscle symptoms (SAMS), ranging from mild myalgia to severe rhabdomyolysis [5]. The onset of these SAMS can lead to patient discomfort, reduced adherence, and, in some cases, discontinuation of therapy, typically emerging within the first 6 months of treatment [6].

On the other hand, regular exercise is well-known to confer improved cardiovascular and metabolic health and reduced morbidity and mortality [7]. However, the interplay between exercise and statin use presents a clinical challenge. Evidence suggests that regular exercise, especially when intense, may exacerbate statin-related muscle symptoms [8–10]. This is particularly concerning for athletes or those following rigorous training regimens, as their increased muscular demand may heighten susceptibility to muscle damage [11]. Studies suggest that moderate exercise does not necessarily worsen SAMS and may even support muscle and metabolic health, indicating a dose-dependent relationship with exercise intensity [12, 13]. Thus, striking the right balance in exercise intensity and frequency maximizes cardiovascular and metabolic benefits while minimizing adverse muscular outcomes in individuals under statin therapy.

Another important dimension to consider when evaluating the interplay between dyslipidemia, statin use, and exercise is its potential impact on the overall quality of life (QoL). QoL encompasses physical health, psychological well-being, social relationships, and environmental factors, all shaped by personal goals, expectations, and cultural contexts through various tools [14, 15]. Regular physical activity correlates positively with QoL, enhancing physical and mental health domains. However, the magnitude and consistency of this relationship vary across different populations and study designs. While some evidence strongly suggests that increased physical activity improves QoL, other findings are less conclusive, highlighting the need for further research [16–20].

Given the complexities inherent in managing patients with dyslipidemia, especially those on statin therapy, understanding the relationship between physical activity levels and QoL in this population is an important clinical and research question. An important clinical and research question is whether higher levels of engagement in physical activity—defined as greater frequency, longer duration, and/or higher intensity of exercise—improve QoL in statin users despite the potential exacerbation of muscle symptoms, or is the benefit counterbalanced by discomfort and reduced adherence? Addressing this question can help clinicians develop tailored exercise recommendations, optimize pharmacological therapy, and improve patient outcomes.

This study aims to assess physical activity levels in patients using statins and determine whether a correlation exists between their physical activity level and overall QoL. By exploring this relationship, we hope to generate insights that may inform more individualized recommendations to optimize both the cardiovascular benefits of statin therapy and patients' QoL, thereby supporting long-term adherence and improved health outcomes.

## Methods

### Ethical approval and study design

This cross-sectional study was part of a specialty thesis project and adhered to the principles outlined in the Declaration of Helsinki. The Non-Interventional Research Ethics Committee of Dokuz Eylül University School of Medicine granted ethical approval (Decision No: 2022/36-02, Date: 9 November 2022). The study aimed to evaluate the relationship between physical activity levels and QoL in adults using statin therapy.

### Study setting and population

The study was conducted between January and June 2023 at the Dokuz Eylül University Family Health Centers. The target population consisted of patients aged 18 years or older registered at these centers and prescribed statin therapy for at least 1 year (*n*: 1326). Patients were approached consecutively as they visited the health centers during the study period, thus minimizing selection bias.

### Inclusion and exclusion criteria

Inclusion criteria were (i) age ≥ 18 years, (ii) current use of any statin medication for ≥1 year, and (iii) willingness to provide informed consent and participate in the study. Exclusion criteria included (i) a diagnosis of major depression or bipolar disorder, (ii) Alzheimer's disease or dementia, (iii) an active cancer diagnosis, and (iv) duration of statin use < 1 year. These exclusions were implemented to avoid confounding influences on QoL and physical activity that could arise from severe psychiatric conditions, cognitive impairment, or active malignancies.

### Sample size determination

The required sample size was calculated using Cochran's formula (*n* = Z²·p·(1−p)/d²), assuming an unknown population proportion. To ensure the maximum sample size, the prevalence was set at 50% with a 95% confidence level and a 5% margin of error, yielding a required sample of 384 participants. Throughout the data collection period, 430 patients with hyperlipidemia presented to the study sites. Of these, 26 patients declined to participate, and 20 did not meet the inclusion criteria (e.g. less than 1 year of statin use, presence of excluded conditions) and were therefore excluded. After these exclusions, 384 eligible patients agreed to participate, meeting the target sample size and ensuring sufficient precision to estimate the relationship between physical activity levels and QoL.

### Data collection procedures

After verbal and written informed consent, participants were interviewed face to face by trained family medicine residents who received standardized questionnaire administration instructions. These interviewers were familiar with the questionnaires and received guidance to minimize interviewer bias and ensure consistent data collection. The data were collected using a personal data form, Turkish versions of the International Physical Activity Questionnaire (IPAQ) long form, and the Short Form-36 (SF-36) Health Survey. The research team developed the personal data form, reviewed it with two experienced family physicians, and pilot-tested it on a small subset of patients (*n* = 10) for clarity. No significant revisions were necessary.

### Measures and instruments

A personal data form was used to collect sociodemographic data [age, sex, education, employment, body mass index (BMI; calculated using measured height and weight)], lifestyle factors (smoking status), and clinical information (presence of chronic conditions, statin type, dose, and duration of use) during data collection.

### IPAQ long form

Developed with the support of the WHO and CDC, the IPAQ long form has been validated in Turkish populations [21, 22]. It assesses work, transportation, household/gardening, leisure time activities, and sitting time. Activities are converted into MET-min/week scores, accounting for frequency, duration, and intensity. Based on total MET-min/week, participants are categorized as inactive, minimally active, or very active. Missing IPAQ data were rare; if a response was missing for a specific activity domain, that domain was excluded from the total MET calculation, and participants were categorized accordingly.

### SF-36 Health Survey

The SF-36, validated in Turkish populations [23, 24], measures eight domains of health-related QoL: physical functioning, role limitations due to physical health, role limitations due to emotional problems, energy/fatigue, emotional well-being, social functioning, pain, and general health perceptions. Higher scores indicate better-perceived health. In accordance with the SF-36 scoring manual, negatively worded items (e.g. item 20 in the Social Functioning domain, where higher responses indicate greater limitations) were reverse-coded so that all items were oriented in the same direction, with higher scores consistently reflecting better functioning. For example, responses 1–5 for item 20 were recoded to 100, 75, 50, 25, and 0, respectively, while responses for item 32 (higher scores indicating fewer limitations) were coded as 0, 25, 50, 75, and 100. Incomplete items were handled per the SF-36 manual guidelines; if more than half of the items in a domain were missing, that domain score was not calculated.

### Statistical analysis

All data were analyzed using IBM SPSS Statistics version 27.0 (IBM Corp., Armonk, NY) and JASP version (0.19.1) (University of Amsterdam, Amsterdam, The Netherlands), with a significance level set at *P* < 0.05. The Kolmogorov–Smirnov test indicated that continuous variables did not follow a normal distribution; thus, nonparametric tests were used. Frequencies and percentages were reported for categorical variables and medians (min–max) for continuous variables. The χ² test compared categorical variables. The Mann–Whitney *U* or Kruskal–Wallis tests were employed for comparisons involving continuous variables and categorical groupings. *Post hoc* comparisons following significant Kruskal–Wallis tests were conducted using Dunn's test with Bonferroni adjustment applied to adjust for multiple comparisons. Spearman's rank correlation explored associations between continuous variables (e.g. MET-min/week and SF-36 domain scores). Potential confounding variables (e.g. age, sex, educational level, comorbidities) were collected and analyzed descriptively. Subgroup differences were evaluated using nonparametric tests. Multivariate analyses were not performed due to the cross-sectional nature and nonparametric distributions. The study's conceptual underpinning—examining how statin use and exercise behaviors impact QoL—guided variable selection.

## Results

### Participant characteristics

A total of 384 patients using statin therapy were included in the study. The mean age was 62.86 ± 10.2 years (median: 64, range: 25–86), and 56.0% of participants were female. Most participants were married (70.8%), had children (87.8%), and were not currently employed (54.2%). Regarding education, 63.3% had completed at least a high school education, and 21.6% reported having income levels exceeding their expenses. Current smoking was reported by 39.8% of participants. The mean body mass index (BMI) was 27.56 ± 3.97 (median: 27.47, range: 18.39–38.22). Hypertension (HT), defined as a previously diagnosed condition under treatment, was present in 75.3% of participants, and diabetes mellitus (DM) was present in 63.8%. Nearly half (47.7%) had a form of cardiovascular disease (CVD), with 35.2% having coronary artery disease. A small proportion had chronic kidney disease (2.9%), psychiatric conditions such as anxiety or mild-to-moderate depression (21.1%), and other chronic illnesses, including hypothyroidism, chronic obstructive pulmonary disease (COPD), and asthma.

Atorvastatin was the most used statin (69.5%), followed by pitavastatin (15.4%) and rosuvastatin (15.1%). Most participants (78.1%) were on moderate-intensity statin therapy, 3.6% received low-intensity treatment, and 18.2% received high-intensity treatment. The median duration of statin use was 5 years (mean: 6.27 ± 4.76, range: 1–19). Table 1 presents an overview of the demographic, clinical, and statin-related characteristics of participants.

**Table 1.** Demographic, clinical, and statin-related characteristics of participants (*n* = 384) (data presented as *n* (%), unless otherwise stated).

Characteristic	Value
**Age (years)** ^a^	62.86 ± 10.2 (64, 25–86)
**Female sex**	215 (56.0)
**Married**	272 (70.8)
**Has children**	337 (87.8)
**Education ≥ high school**	290 (75.5)
**Currently unemployed**	208 (54.2)
**Income ≥ expenses**	267 (69.5)
**Current smoker**	153 (39.8)
**BMI (kg/m²)^a^**	27.56 ± 3.97 (27.47,18.39–38.22)
**Hypertension (HT)**	289 (75.3)
**Diabetes mellitus (DM)**	245 (63.8)
**Any cardiovascular disease**	183 (47.7)
**Chronic kidney disease**	11 (2.9)
**Anxiety/depression (mild–moderate)**	81 (21.1)
**Atorvastatin use**	267 (69.5)
**Rosuvastatin use**	58 (15.1)
**Pitavastatin use**	59 (15.4)
**Moderate-intensity statin**	300 (78.1)
**Statin use (years)** ^a^	6.27 ± 4.76 (5.1–19)

^a^Mean ± SD (median, range).

### Physical activity levels

Based on IPAQ categorizations, 18.2% of participants were inactive, 65.1% were minimally active, and 16.7% were very active.

### QoL scores (SF-36)

Median scores for SF-36 domains indicated moderate levels of health-related QoL. For instance, the median (range) scores were physical functioning 70 (10–100), role limitations due to physical health 50 (0–100), role limitations due to emotional problems 100 (0–100), energy/fatigue 50 (5–85), emotional well-being 68 (12–100), social functioning 75 (0–100), pain 67.5 (10–100), and general health 40 (0–90).

### Differences in SF-36 QoL domains according to physical activity levels

Kruskal–Wallis tests showed that all SF-36 domains—except role limitations due to emotional problems—significantly differed across the three physical activity categories (*P* < 0.001 for all significant domains). *Post hoc* pairwise comparisons following Kruskal–Wallis tests were conducted using Dunn's test with Bonferroni adjustment, Idicating that participants in the very active group had significantly higher scores in physical functioning, role limitations due to physical health, energy/fatigue, social functioning, and general health perceptions compared to those in the inactive and minimally active groups (Supplementary Table S1). Similarly, minimally active students scored significantly higher than the inactive group in these domains. Both very active and minimally active groups outperformed the inactive group in the pain and emotional well-being domains. Still, there were no significant differences between the very active and minimally active groups. Table 2 summarizes the SF-36 domain scores by physical activity category.

**Table 2.** SF-36 domain scores by PA category [median (min–max) scores and *P*-values from Kruskal–Wallis tests. *Post hoc* comparisons indicated that very active > minimal active > inactive for all significant domains except emotional role limitations].

Domain	Inactive (*n* = 70)	Minimal active (*n* = 250)	Very active (*n* = 64)	*P*-value
**Physical functioning**	30 (10–95)	70 (25–100)	90 (25–100)	<0.001
**Role limitations due to physical health**	0 (0–75)	50 (0–100)	100 (0–100)	<0.001
**Role limitations due to emotional problems**	100 (0–100)	100 (0–100)	100 (0–100)	0.384
**Energy/fatigue**	25 (5–85)	50 (10–85)	60 (20–80)	<0.001
**Emotional well-being**	52 (28–88)	72 (40–96)	76 (40–96)	<0.001^a^
**Social functioning**	25 (10–87.5)	75 (25–100)	75.5 (50–100)	<0.001
**Pain**	45 (10–100)	67.5 (10–100)	67.5 (22.5–100)	<0.001^a^
**General health**	20 (10–80)	45 (20–90)	65 (20–90)	<0.001

^a^ *Post hoc* comparisons were not significant between the very active and minimally active groups.

### Correlations between physical activity (MET-min/week) and SF-36 domains

Spearman's correlation analyses revealed moderate positive correlations between MET-min/week and several SF-36 domains. The strongest correlations were observed with physical functioning (*r* = 0.700, *P* < 0.001), social functioning (*r* = 0.598, *P* < 0.001), role limitations due to physical health (*r* = 0.567, *P* < 0.001), and general health (*r* = 0.546, *P* < 0.001). Energy/fatigue (*r* = 0.509, *P* < 0.001), emotional well-being (r = 0.332, *P* < 0.001), and pain (*r* = 0.345, *P* < 0.001) also showed significant positive correlations. No significant correlation was found for role limitations due to emotional problems (*P* > 0.05). Table 3 presents the correlation between physical activity and SF-36 Domains.

**Table 3.** Correlation between PA (MET-min/week) and SF-36 domains.

Domain	*r*	*P*-value
**Physical functioning**	0.700	<0.001
**Role limitations due to physical health**	0.567	<0.001
**Role limitations due to emotional problems**	0.086	0.093
**Energy/fatigue**	0.509	<0.001
**Mental health**	0.332	0.001
**Social functioning**	0.598	<0.001
**Pain**	0.345	<0.001
**General health**	0.546	<0.001

Table 4 presents the correlation coefficients between sitting duration and SF-36 domains. The results indicate a significant negative relationship between sitting duration and physical function, role limitations due to physical health, energy/fatigue, emotional well-being, social functioning, pain, and general health. Notably, higher sitting duration is associated with reduced physical and mental well-being, particularly in energy/fatigue (Spearman's rho = −0.556, *P* < 0.001) and bodily function (Spearman's rho = −0.504, *P* < 0.001). While weaker, the association with role limitations due to emotional problems remains statistically significant (*P* = 0.039).

**Table 4.** Correlation between sitting duration and health-related outcomes.

Health-related variable	Spearman's rho (correlation coefficient)	*P*-value
**Physical function**	−0.504	<0.001
**Role limitations due to physical health**	−0.417	<0.001
**Role limitations due to emotional problems**	−0.106	0.039
**Energy/fatigue**	−0.556	<0.001
**Mental health**	−0.441	<0.001
**Social functioning**	−0.529	<0.001
**Pain**	−0.371	<0.001
**General health**	−0.486	<0.001

## Discussion

Dyslipidemia has a prevalence of 36.6%, according to a comprehensive meta-analysis in Turkey, indicating a common health issue and that cardiovascular risk factors are widespread in the country [3]. Its prevalence may also negatively affect QoL. Regular physical activity (PA) improves energy levels, mood, and social interactions and enhances overall QoL [17]. Encouraging PA to manage dyslipidemia is a key strategy for improving patients' QoL. Regular PA enhances insulin sensitivity, helps control body weight, reduces CVD risk, and can favorably modify lipid profiles by increasing high-density lipoprotein cholesterol (HDL-C) and decreasing LDL-C and triglyceride levels. Although lifestyle modifications are foundational in dyslipidemia management, pharmacotherapy, particularly statins, remains central to lowering LDL-C [25].

However, the interaction between statin use and PA can be complex; statins may cause musculoskeletal side effects that could potentially reduce adherence to therapy and impair QoL [26]. Conversely, regular and appropriately tailored PA may mitigate statin-related side effects and enhance overall health benefits [12]. Thus, PA type, duration, and intensity in patients taking statins should be carefully planned.

### Sociodemographic characteristics and comorbid conditions

This study included 384 participants from a primary care setting. Most were female (56.0%), with a mean age of 62.86 ± 10.28 years (median: 64). Approximately 40% were current smokers, and the majority were overweight (42.4%). Compared with broader samples, such as those in meta-analyses focusing on statin use, participants in this study were older and more frequently female [27, 28]. The higher age and female predominance may reflect the utilization patterns of primary care services, with older and female patients visiting more regularly and the relatively more straightforward access and availability of these clinics to such populations. Additionally, this study's participants had higher BMIs and a more significant burden of comorbidities than the general population, aligning with the profile of individuals likely to be prescribed statins due to elevated cardiovascular risk [28, 29]. Furthermore, most participants had HT, DM, and CVD, which is consistent with previous research reflecting a complex clinical profile with multiple cardiovascular risk factors [27, 28].

### PA and QoL

In this study, 18.2% of participants were inactive, 65.1% were minimally active, and 16.7% were very active. Various sociodemographic, economic, educational, and health-related factors may contribute to this distribution of PA levels [30].

When comparing SF-36 scores to established Turkish norms [24], statin users in our study displayed lower physical functioning and general health perceptions, likely influenced by older age, higher BMI, and more frequent comorbidities. Although we expected that high-intensity PA might exacerbate statin-related muscle symptoms and thereby negatively affect QoL, our findings were consistent with previous literature demonstrating that higher levels of PA are generally associated with better QoL parameters [8, 9]. Our data showed that active participants had significantly higher scores in physical functioning, role limitations due to physical health, energy/fatigue, social functioning, and general health than other groups. Minimally active participants also scored higher than the inactive group in these domains. Very active and minimally active groups scored significantly higher than inactive groups for pain and emotional well-being. Only role limitations due to emotional problems showed no significant difference between groups. These results support the notion that increasing levels of PA are positively associated with multiple QoL domains.

While no previous studies have focused explicitly on PA–QoL relationships in statin users among primary care patients, our findings align with general trends observed in older populations. A systematic review that examined studies with an average age over 60 found positive associations between PA and various aspects of QoL, including functional capacity, general QoL, emotional well-being, and energy/fatigue [31]. Our results suggest that despite statins' potential muscle-related side effects, regular and appropriately guided PA may still provide significant QoL benefits.

One reason we did not observe a detrimental effect from intense PA on QoL in statin users might be that SAMS is expected to occur in statin users regardless of PA, usually within the first 6 months of therapy [7]. What we aimed to assess was whether, in long-term statin users (≥1 year), increasing PA intensity would lead to a recurrence of SAMS, like symptoms such as myalgias, and a subsequent decline in QoL. However, no such detrimental effect was observed.

The significant negative correlations between sitting duration and health-related outcomes, such as physical function, energy levels, mental health, and social functioning, highlight the detrimental effects of sedentary behavior on overall health. Particularly notable is the strong association with reduced energy and vitality. This suggests prolonged sitting diminishes perceived energy levels, potentially creating a cycle where low energy discourages PA and perpetuates inactivity [32].

The link between sitting duration and physical function underscores the risks of decreased mobility, especially among older adults, emphasizing the need for interventions to reduce sedentary time and promote PA [33].

Similarly, the negative impact on mental health and social functioning highlights how prolonged sitting may contribute to isolation, anxiety, and reduced QoL, further limiting individuals' ability to engage in emotional and social roles effectively [34].

### Limitations

Unmeasured confounders might influence both PA and QoL. Although we used validated questionnaires (IPAQ and SF-36), numerous factors can influence QoL. The presence of multiple comorbidities, differing sociodemographic profiles, and various statin types and dosages complicate generalizability. Variations in statin types and dosage may influence both lipid control and the extent of side effects, potentially affecting PA levels and QoL outcomes. As dosage information was not analyzed in detail, this remains a limitation of the study. Although no participants with diagnosed neurological or muscular disorders were included, varying levels of physical limitation were possible among participants. While this variability may have influenced engagement in PA, it reflects real-world clinical diversity. SAMS were not directly assessed in this study. Although the SF-36 pain domain may reflect musculoskeletal discomfort to some extent, it does not specifically measure SAMS. Additionally, all patients had been on statins for at least 1 year, so the statin-related side effects that might have occurred rarely occurred.

## Conclusions and recommendations

To our knowledge, this is among the first studies to explore the specific association between PA levels and QOL in statin users, conducted among primary care patients. Thus, it contributes novel insights to an underexplored area. Except for role limitations due to emotional problems, all QoL domains were positively associated with PA. This finding reinforces the importance of encouraging PA as part of lifestyle modification in this population.

Future studies should be observational and longitudinal to better understand the complex interplay between statin use, PA, and QoL. Given the high prevalence of comorbid conditions and continued smoking in these patients—factors that further escalate cardiovascular risk—primary care interventions should emphasize comprehensive risk factor management, including smoking cessation and promotion of regular PA.

## Supplementary Material

cmaf080_Supplementary_Data

## Data Availability

The data supporting the findings of this study are available upon reasonable request from the corresponding author. Due to ethical considerations and participant confidentiality, data cannot be shared publicly.
