# Supplementary material for: Physical activity and quality of life in statin users
Source: Fam Pract. 2025 Oct 21;42(6):cmaf080. doi: 10.1093/fampra/cmaf080 (PMC12539343; doi:10.1093/fampra/cmaf080)
Supplement: cmaf080_Supplementary_Data [file cmaf080_supplementary_data.pdf]

Supplementary Table 1. Dunn's Post Hoc Comparisons of Physical Activity Groups on SF-36 Domains

Domain	Comparison	z	W_i	W_j	r_{β}	p	pBonf	pHolm
Physical Functioning	Inactive – Minimal Active	−8.178	76.014	198.452	0.701	< .001	< .001	< .001
	Inactive – Very Active	–11.523	76.014	296.656	0.902	< .001	< .001	< .001
	Minimal Active – Very Active	−6.332	198.452	296.656	0.581	< .001	< .001	< .001
Role Limitations – Physical	Inactive – Minimal Active	−7.665	88.907	201.396	0.622	< .001	< .001	< .001
	Inactive – Very Active	−9.705	88.907	271.055	0.806	< .001	< .001	< .001
	Minimal Active – Very Active	−4.582	201.396	271.055	0.403	< .001	< .001	< .001
Role Limitations – Emotional	Inactive – Minimal Active	−1.295	179.386	194.914	0.080	0.195	0.586	0.586
	Inactive – Very Active	−1.176	179.386	197.414	0.098	0.240	0.719	0.586
	Minimal Active – Very Active	−0.201	194.914	197.414	0.012	0.840	1.000	0.840
Energy/Fatigue	Inactive – Minimal Active	−7.720	92.193	207.718	0.636	< .001	< .001	< .001
	Inactive – Very Active	−7.867	92.193	242.766	0.651	< .001	< .001	< .001
	Minimal Active – Very Active	−2.261	207.718	242.766	0.220	0.024	0.071	0.024
Emotional Well-being	Inactive – Minimal Active	−6.402	114.164	209.922	0.514	< .001	< .001	< .001
	Inactive – Very Active	−5.016	114.164	210.125	0.440	< .001	< .001	< .001
	Minimal Active – Very Active	−0.013	209.922	210.125	0.018	0.990	1.000	0.990
Social Functioning	Inactive – Minimal Active	−8.309	81.543	202.570	0.668	< .001	< .001	< .001
	Inactive – Very Active	–10.359	81.543	274.523	0.859	< .001	< .001	< .001
	Minimal Active – Very Active	−4.769	202.570	274.523	0.416	< .001	< .001	< .001

Pain	Inactive – Minimal Active	−6.125	116.236	207.554	0.473	< .001	< .001	< .001
	Inactive – Very Active	−5.290	116.236	217.109	0.534	< .001	< .001	< .001
	Minimal Active – Very Active	−0.619	207.554	217.109	0.047	0.536	1.000	0.536
General Health	Inactive – Minimal Active	−7.372	91.664	201.996	0.608	< .001	< .001	< .001
	Inactive – Very Active	−9.092	91.664	265.695	0.775	< .001	< .001	< .001
	Minimal Active – Very Active	−4.108	201.996	265.695	0.369	< .001	< .001	< .001
